# Curiosity in Online Video Concept Learning and Short-Term Outcomes in Blended Medical Education

**DOI:** 10.3389/fmed.2021.772956

**Published:** 2021-11-05

**Authors:** Cheng-Maw Ho, Chi-Chuan Yeh, Jann-Yuan Wang, Rey-Heng Hu, Po-Huang Lee

**Affiliations:** ^1^Department of Surgery, National Taiwan University Hospital, Taipei, Taiwan; ^2^School of Medicine, College of Medicine, National Taiwan University, Taipei, Taiwan; ^3^Department of Medical Education, National Taiwan University Hospital, Taipei, Taiwan; ^4^Department of Internal Medicine, National Taiwan University Hospital, Taipei, Taiwan; ^5^Center of Faculty Development and Curriculum Integration, College of Medicine, National Taiwan University Hospital, Taipei, Taiwan; ^6^Department of Surgery, E-Da Hospital, I-Shou University, Kaohsiung, Taiwan

**Keywords:** online video learning, curiosity, concept, learning outcome, medical education

## Abstract

**Background:** A student's level of curiosity in a subject after learning about it through online videos has not been addressed well in the medical education field. The purpose of this study, therefore, was to investigate online learning's effect on the stimulation of curiosity and short-term learning outcomes in a blended framework of precision medical education.

**Methods:** A mixed-methods research design was used. During the 2020 academic year, all fifth-year medical students who, prior to class, viewed 6 video clips that presented 6 core concepts were invited to complete a survey and self-reflection on their learning process to assess their level of curiosity in each concept. For each group of medical students, teaching assistants helped collect anonymous survey data and summative assessment scores representing the students' learning outcomes. Video-viewing patterns, attained through an action log transformation, were also coded for analysis. Mann–Whitney *U* and Kruskal–Wallis tests were employed to compare differences between groups, and multiple linear regression was used to select the factors affecting learning outcomes. Qualitative data were content-coded through a descriptive approach using thematic analysis.

**Results:** Of 142 medical students, 136 watched the online videos, 124 responded to the questionnaires, and 92 provided comments. Students' curiosity levels after learning about each concept through online videos significantly correlated with the degree to which a concept was learned. Medical students spent a median of 1.6 h online, and pause frequency correlated with curiosity in certain concepts. Aroused curiosity was associated with short-term learning outcomes in inconsistent effect sizes and directions. Students' feedback revealed various dimensions of curiosity, including novelty acknowledgment, recognition of an information gap, and information-seeking requests.

**Conclusions:** Curiosity can be induced through online video learning platforms and has a role in short-term learning outcomes in medical education.

## Introduction

Curiosity can be broadly defined as the desire to acquire new knowledge and new sensory experiences, which motivates exploratory behavior (1–3). An individual's degree of curiosity varies according to their personality traits (2) and can be independently induced; for example, two people might be drawn to different aspects of the same stimulus (2, 3). In the medical education field, studies in cognitive psychology and education have suggested that common instructional practices may inadvertently suppress curiosity by conflating haste with efficiency, neglecting negative emotions, promoting overconfidence, and using teaching approaches that encourage passive learning (4). Attributes of the instructor that contribute to the development of a student's curiosity include patience, a habit of inquiry, emotional candor, intellectual humility, transparency, and recognition of the benefits of learning from peers (4). Specific educational strategies that can support curiosity in both classroom and clinical settings include the mindful pacing of teaching, modeling effective control of emotions, confronting uncertainty and overconfidence, using inquiry-based learning, helping students see familiar situations as novel, simultaneously contemplating multiple perspectives, and maximizing the value of small-group discussions (4).

## Theoretical Background and Importance of Curiosity in Medical Education

George Loewenstein described curiosity as “a cognitive induced deprivation that arises from the perception of a gap in knowledge and understanding” (2). Epistemic curiosity is “the desire for knowledge that motivates individuals to learn new ideas, eliminate information gaps, and solve intellectual problems” (5). The development of deliberate, focused, and sustained epistemic curiosity should, therefore, be a core element of teaching and learning in medical education (6). Moreover, calls for self-reflection, critical thinking, and teamwork are meaningless in the absence of curiosity (4). Kidd and Hayden expanded information gap theory by proposing that studying the motivation behind information-seeking behavior in its ethological context is more productive than defining curiosity itself (7). This conceptualization indicates that online video learning using a threshold concept strategy (8) presents various opportunities for medical students to develop epistemic curiosity.

Although the importance of curiosity in medical education was perceived and critically reminded in literature for a long time (4, 9–12), relevant evidence was accumulated sluggishly. Stenernszus et al. found that trait curiosity (individual characteristic) is relatively stable across a 4-year undergraduate program of medical education whereas there is more variability in state curiosity (arousal of curiosity by the educational context), which is consistently lower than trait curiosity in each year (13). Medical students' state curiosity may not be optimally supported in the environment of medical education (13). Richards et al. demonstrated that students with high levels of trait curiosity tended to use learning strategies that promoted understanding rather than memorization (14). The need to evoke curiosity in medical education is highlighted, especially in the current setting of teaching which relies heavily on online learning and online video materials have become an integral part of instruction at universities.

We previously explored the possibility of practicing precision medical education based on cognitive load theory and the theory of multimedia learning (15–19). We summarized that implementing precision medical education in the blended medical education is feasible and online video learning is an ideal platform for balancing the dilemma between increasing cognitive load of class content and practice of precision medical education (15). In addition, inverse concept change was frequently documented among participants and students' feedback of online video learning experiences revealed aroused study interest and motivation (15). These observations were subtle signs of aroused curiosity which trigger us for further study. Therefore, we hypothesized that this instructional design could stimulate students' curiosity for online video learning of these concepts. This study examined students' levels of curiosity arousal after online video learning and investigated the association of curiosity with short-term learning outcomes.

## Methods

### Research Type, Context, and Participants

**Figure 1:**
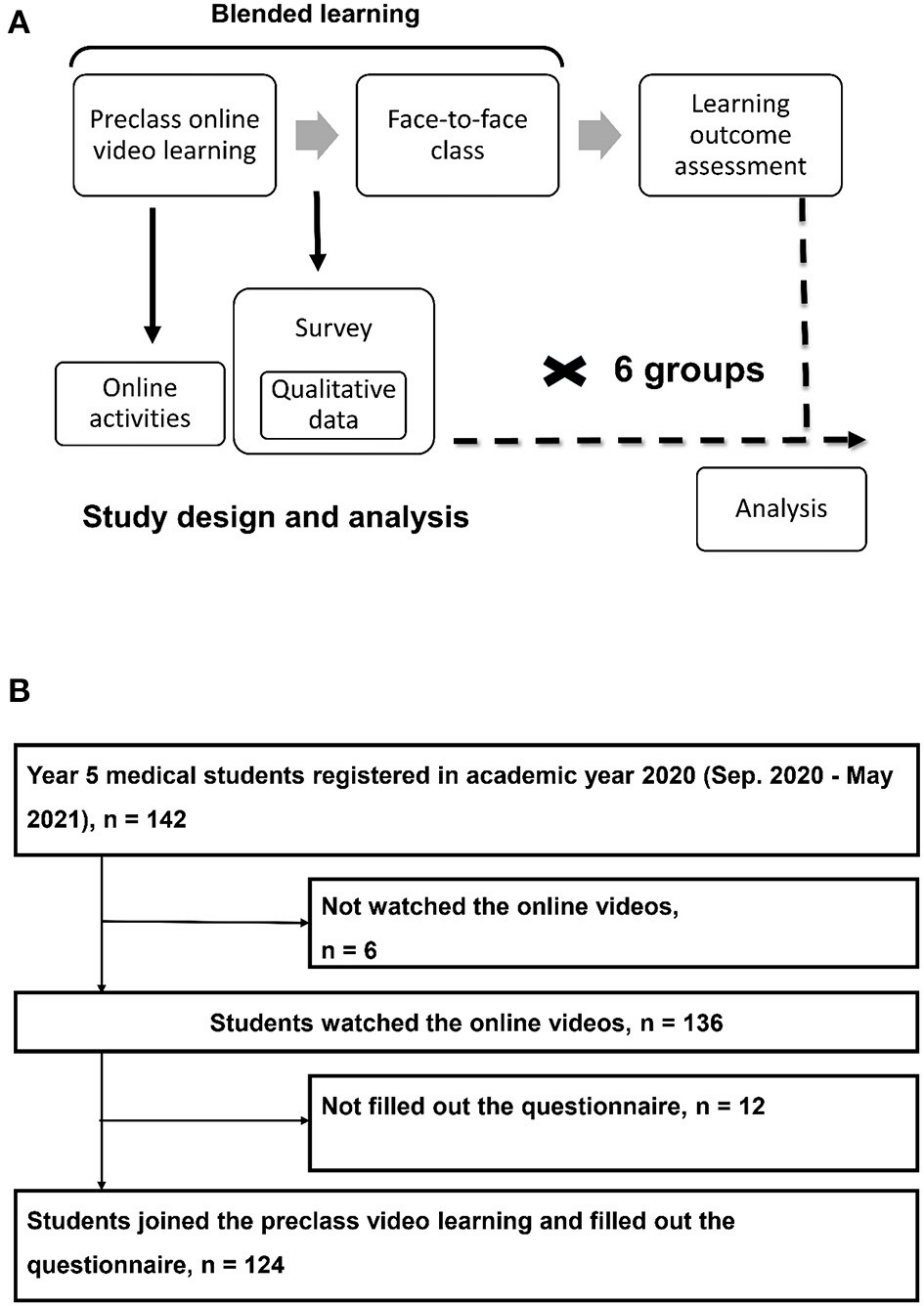
Study design **(A)** and participants **(B)**.

This study involved a survey using cross-sectional mixed-methods study design (20). The survey consisted of questionnaires (quantitative part) and free comments (if any, qualitative part) (Supplementary Table 1). The qualitative component of this study was embedded to enhance a largely quantitative study (Figure 1A) (21). This embedded mixing facilitates quantitative and qualitative analyses to complement each other (21). Clinical teaching on the subject of acute liver failure is a section of a core compulsory course (including 23 sections) for surgery students in their first clinical (fifth) year (8). Almost all students were between 23 and 24 years of age, with few post-baccalaureate students in every school year. Each course section consists of a 1-h class, with 22–24 medical students enrolled in each round. Six rounds of teaching are conducted in 1 academic year at National Taiwan University Hospital. Between September 2020 and May 2021, 142 fifth-year medical students took the compulsory core course on surgery, and they were invited to participate in this survey. The Institutional Review Board of National Taiwan University Hospital approved this study as an exempt protocol (201809078W and 202006048W). Participation in the survey was considered implied consent from the participants.

The curriculum development committee assigned HCM to develop the curriculum for the acute liver failure section. HCM summarized 6 threshold concepts of acute liver failure according to the educational goals of the curriculum development committee and incorporated them into the course design and practice (8). A blended course section of acute liver failure was initiated and practiced beginning in 2018 (8, 15).

The course content was divided into two stages: pre-class online video learning and face-to-face classroom instruction. When redesigning the classic course into a blended one, consideration was specifically given to dividing the learning materials in a manner such that they would retain a stimulating effect and be integrated at the end of the learning journey. The instructional methods for the online video portion of the course were based on the coherence (excluding extraneous material) and segmenting (message is presented in user-paced segments rather than as a continuous unit) principles of Mayer's multimedia learning (18, 19).

Previously, a 10-min online video for pre-class learning was developed to minimize the extraneous load by removing non-essential content, breaking content into smaller segments, and enabling learners to control the pace (15). In the 2020–2021 academic year, one 10-min online video was remade into 6 video clips, each lasting <2 min, for six individual concepts. A title with a short summary of each concept was added to arouse interest and curiosity. The clinical teacher (HCM) selected and uploaded the updated review literature to the webpage to make available optional additional reading. A list of chart numbers was provided on the intranet for a real-world clinical case analysis.

At the beginning of the surgery course, students were instructed to watch the online videos explaining the core concepts before the face-to-face class. Subsequently, they were free to respond to an online questionnaire (Supplementary Table 1) in the university intranet (22). Changes in the understanding of concepts were rated on a 5-point Likert scale (“totally changed,” “largely changed,” “changed and unchanged in equal measure,” “mostly unchanged,” and “totally unchanged”). The following categories were evaluated: curiosity induced in individual concepts; concepts that motivated medical students to learn more; concepts requiring further clarification during face-to-face classes; loading, difficulty, and satisfaction of online video learning prior to class; class style (teaching method) expectations for the upcoming face-to-face class; and comments or questions.

The survey listed the four class styles: complete and thorough introduction (subsequently referred to as thorough), concept orientation to stimulate study interest (concept), discussions between the teacher and students creating a learning experience (discussion), and self-learning and class presentations (presentation) (15). The teacher developed the face-to-face class style for each round of students based on the survey responses (15).

The outcome measurements in this study consisted of two summative assessments. The first was a written exam [total possible score: 100 marks (points)] taken at the end of the surgery course round, which included 3 points for a short essay question concerning the acute liver failure section. The second assessment was a clinical case-based analysis (total possible score: 100 marks that contribute to grade point average for the section course) that was submitted online prior to the completion of the surgery course. The clinical teacher (HCM) graded the medical students' work after they completed the surgery course.

### Online Learning Activities and Patterns of Online Video-Viewing Behaviors

Cumulative website page views, webpage visit/browsing durations, and action logs of video viewing for each medical student were documented anonymously in the management platform at the end of the school year. The action logs of video viewing were transformed into visualization plots containing intensity (defined as peak times of viewing with a duration lasting more than 2 s), extensity (completeness, categorized as 1 of 3 types: viewing <50%, 50–90%, or more than 90% of the video), and pause frequency (the number of times the video was paused). HCM and WJY independently performed pattern coding and eventually reached consensus.

### Data Collection

For each round of students, administrative teaching assistant (SKW) helped collect anonymous survey data before the class.

Demographic data, scores, and online activities, including action logs of the online video viewing, total webpage view counts, and webpage visit durations, were anonymously collected at the end of the school year by administrative teaching assistants.

### Qualitative Data Analysis

Student comments were independently content-coded through a descriptive approach using thematic analysis (8, 15, 23) by HCM and WJY, who eventually reached consensus. Codes corresponding to learning experiences were previously developed by a team comprising a surgeon specialist (HCM), an experienced medical education specialist (YCC), and an administrative researcher (WJY) (15). Regular meetings were held to discuss and resolve all coding discrepancies and to combine codes (15). According to the survey responses in each round of students, the teacher in charge (HCM) answered questions posed by students, validated the codes that reflected their opinions through anonymous discussions in class, and adjusted the class style (15).

At the end of the school year, identified themes were combined and compared to generate a final set representing the range of student feedback on the online video learning process. The research questions were to identify clues of induced curiosity, learning interests, and self-reported learning outcomes, in order to complement the quantitative analysis. Each student comment could include several codes across various categories (general, infrastructure, curiosity, learning outcome, and miscellaneous).

### Quantitative Data Analysis

Quantitative data were expressed as means, medians, or percentages, where appropriate. Scores were compared using Student's *t* test. Non-parametric tests were employed to compare group differences other than scores. The Mann–Whitney *U* and the Kruskal–Wallis tests were employed to compare the differences in continuous variables among quantitative outcome variables for 2 and 3 groups, respectively. Kendall's tau coefficient was used to measure the ordinal association between 2 measured quantities. The multiple general linear regression model with a backward elimination method was used to select potential factors associated with the assessment scores. A two-sided *P* < 0.05 was considered statistically significant. Statistical analyses were performed using SPSS version 21.0 (SPSS Inc., Chicago, IL, USA).

## Results

### Demographics and Curiosity Spectrum

Of the 136 fifth-year medical students (136/142, 95.8%) who participated in the online video learning, 124 (124/136, 91.2%) completed the survey and were enrolled in the study (Figure 1B). These participants were predominantly male (75%), felt “just fine” about the work load and difficulty of online video learning (82.3 and 90.3%), were satisfied with online learning (91.9%), and provided comments (74.2%; Table 1). More than half of the participants preferred a class style of “through” (55.6%). Participants spent an average of 1.6 h [interquartile range (IQR), 3720.5-8634.8 s] watching the videos, and the median number of webpage visits was 194.5 (144.5–263.8). The summative assessment results of short-term learning outcomes indicated an average score of 87.9 ± 12.4 for the clinical case-based analysis and 2.4 ± 0.9 for the essay question.

**Table 1:** Characteristics of Participants Taking Surveys (Mann–Whitney *U* or chi-square test).

		All, *n* = 124^*^	**Focused curiosity, *n* = 63**	**Diverse curiosity, *n* = 59**	***P***
Gender					0.684
Male (%)		93 (75)	48 (76.1)	43 (72.9)	
Female (%)		31 (25)	15 (23.8)	16 (27.1)	
Group					0.284
Semester 1	1	19	12	6	
	2	23	12	11	
	3	23	14	9	
Semester 2	4	20	6	14	
	5	22	10	11	
	6	17	9	8	
Webpage view counts, median (IQR)		194.5 (144.5–263.8)	172.0 (134.0–246.0)	201.0 (179.0–291.0)	0.031
Visit durations (sec), median (IQR)		5602.5 (3720.5–8634.8)	5597.0 (2767.0–9738.0)	5705.0 (3638.0–8616.0)	0.612
**Subjective**					
Loading of online video learning					0.254
Heavy (%)		8 (6.5)	3 (4.8)	5 (8.5)	
Just fine (%)		102 (82.3)	55 (87.3)	45 (76.3)	
Light (%)		13 (10.5)	5 (7.9)	8 (13.6)	
Difficulty					0.478
Hard (%)		3 (2.4)	2 (3.2)	1 (1.7)	
Just fine (%)		113 (90.3)	59 (93.7)	52 (88.1)	
Easy (%)		7 (5.6)	2 (3.2)	5 (8.5)	
Satisfaction					0.570
Satisfied (%)		114 (91.9)	58 (92.1)	55 (93.2)	
So so (%)		9 (7.3)	5 (7.9)	3 (5.1)	
Class style preference					0.146
Through (%)		69 (55.6)	31 (49.2)	36 (61.0)	
Concept (%)		36 (29.0)	21 (33.3)	15 (25.4)	
Discussion (%)		18 (14.5)	11 (17.5)	7 (11.9)	
Providing comments (%)		92 (74.2)	46 (73.0)	45 (76.3)	0.835
Overall assessment					
**Objective scores**					
Case analysis, mean (SD)		87.9/100 (12.4)	89.1 (4.5)	86.4 (16.8)	0.226^∧^
Essay question, mean (SD)		2.4/3 (0.9)	2.5 (0.9)	2.3 (0.9)	0.484^∧^

* *2 participants did not complete the survey item on curiosity*.

∧ *Student's t test*.

*IQR, interquartile range; SD, standard deviation*.

**Figure 2:**
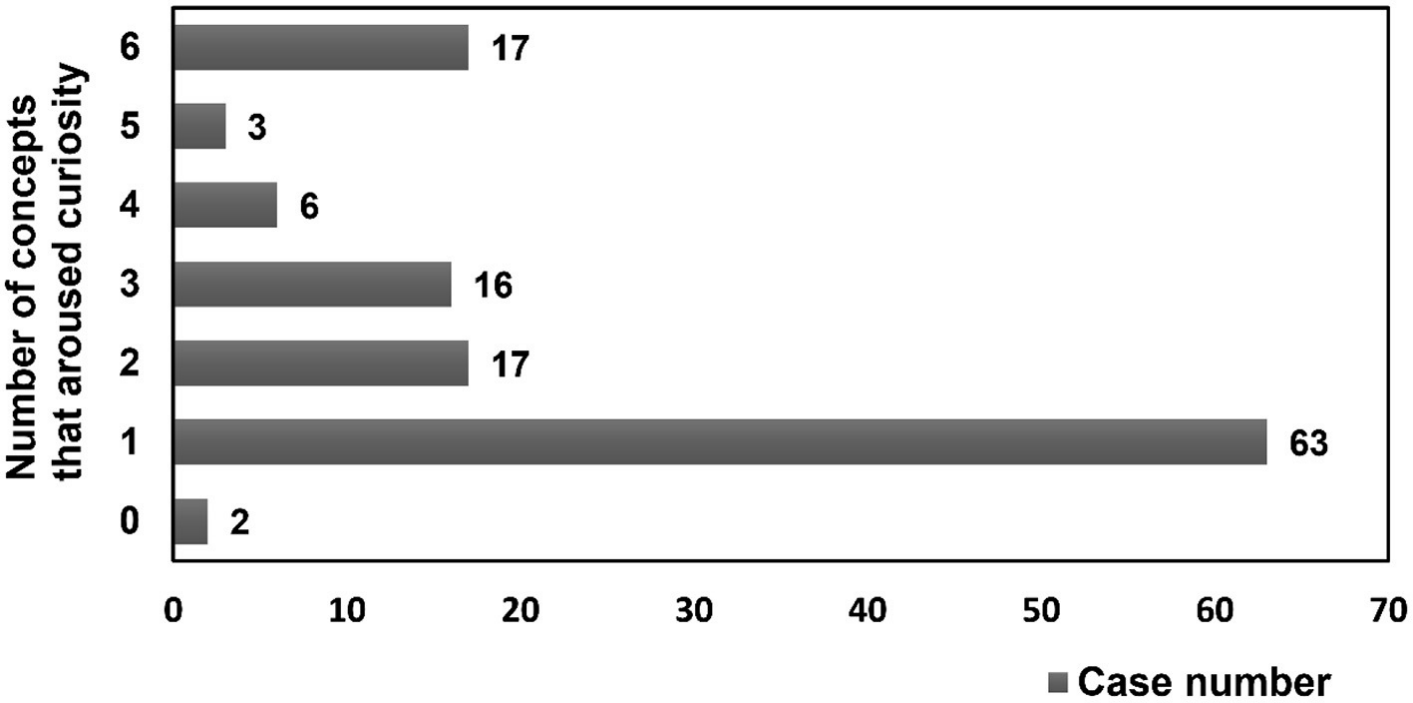
Number of concepts that aroused curiosity in participants.

Half of the participants (63/124, 50.8%) reported curiosity in only 1 concept after online video learning, and 59 participants expressed curiosity for 2–6 concepts (Figure 2). Two participants did not report feeling curious about any concept. Focused (only 1 concept) and diverse (more than 1 concepts) curiosity did not significantly differ when compared between genders, groups, durations of time spent online, perceptions of loading, perceptions of difficulty, perceptions of satisfaction with video learning, class preferences, whether comments were provided, and learning assessments but did significantly differ for accumulated webpage view counts [focused vs. diverse: 172.0 (134.0–246.0) and 201.0 (179.0–291.0); Table 1].

### Online Video-Viewing Patterns

**Figure 3:**
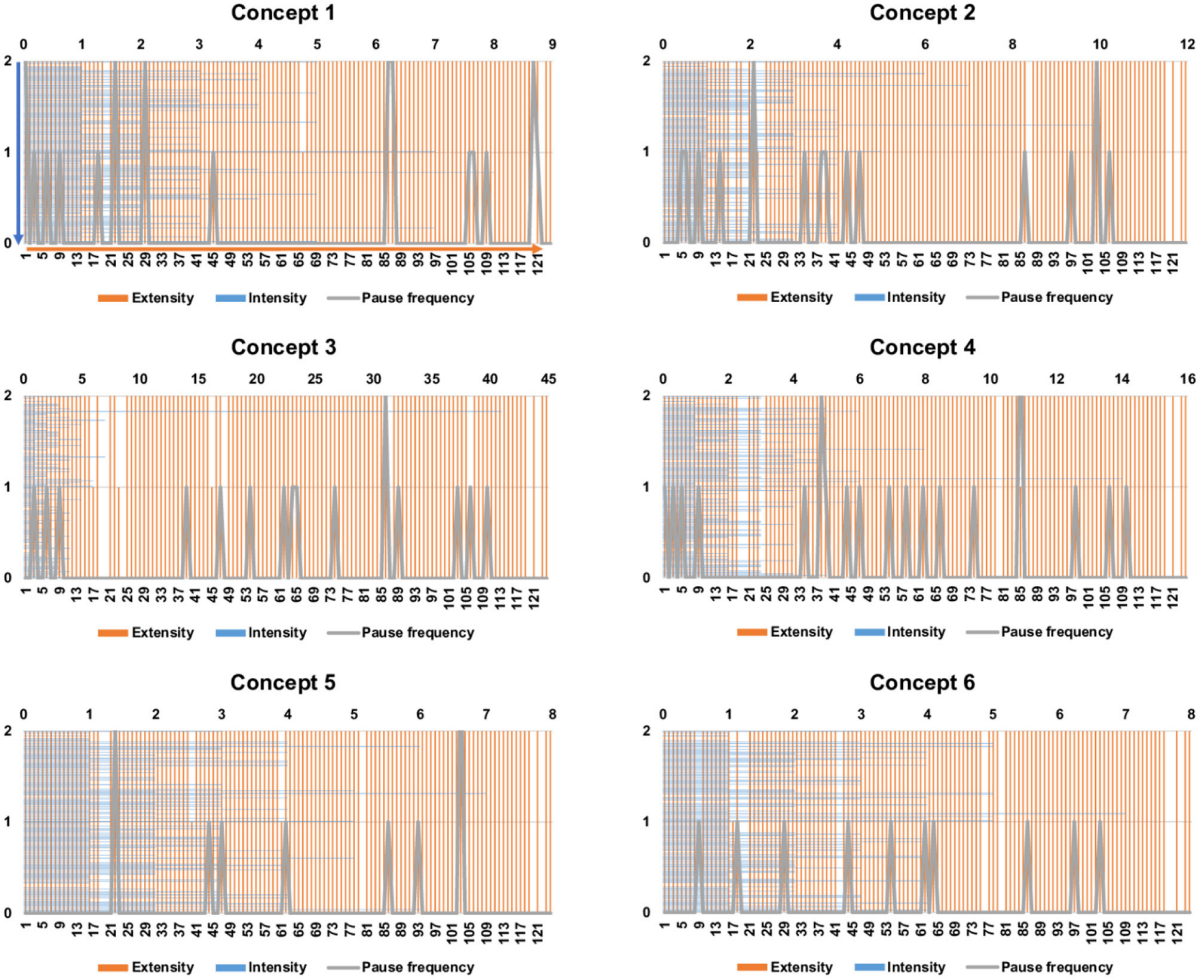
Pre-class online video-viewing patterns (intensity, extensity, pause). (Upper X axis) Intensity (viewing times): Each blue line represents a case. Arrow shows the direction of cumulative cases. (Y axis, orange) Extensity (content completeness of video watching): 0, <50%; 1, 50–90%; 2, more than 90%. (Y axis, gray) Pause frequency during online video learning in each case. (Lower X axis): case number for extensity and pause frequency. Arrow shows the direction of cumulative cases.

The intensity, extensity, and pause frequency of online video-viewing patterns for 6 concepts among 124 participants are shown in Figure 3. Most participants watched more than 90% of the 6 online videos. The median values for the intensity pattern were between 50 and 90% for the six videos. Total pauses when viewing online videos for concepts 1 −6 were 22, 16, 16, 21, 10, and 10, respectively (Figure 3). Statistically, the patterns between the 6 online videos clips were nonsignificant in terms of intensity (*P* = 0.082), extensity (*P* = 0.626), and pause frequency (*P* = 0.239, Kruskal–Wallis test).

### Correlation of Curiosity With Other Parameters

Table 2 displays the correlation of curiosity with other parameters in 6 core concepts. Positive significant correlations between the “most learnt” concepts and their specific curiosities were noted for all 6 concepts. Other self-reported parameters positively correlated with specific curiosities were noted for “expect more” (concept 4) and “teach more” (concept 4) and negatively for “agree with previous understanding” (concept 2 and concept 3; Table 2A). For objective video-viewing patterns, curiosity significantly correlated with extensity (concept 3) and pause frequency (concept 5; Table 2B). Borderline significance was noted in pause frequency for concepts 1 and 4 (*P* = 0.061 and *P* = 0.072).

**Table 2:** Kendall's Tau-B correlation coefficient of curiosity with other parameters.

	**Curious concept**	**Most learnt concept**	***P***	**Expect more**	***P***	**Teach more**	***P***	**Concept agreed with previous thought**	***P***
**(A) Self-reported factors**									
C1	31	0.478	<0.001	0.114	0.205	0.013	0.886	0.013	0.882
C2	61	0.344	<0.001	0.063	0.488	0.081	0.371	−0.205	0.013
C3	58	0.405	<0.001	0.069	0.446	−0.069	0.445	−0.187	0.026
C4	52	0.292	0.001	0.200	0.027	0.240	0.008	−0.118	0.154
C5	47	0.216	0.017	0.145	0.107	0.091	0.312	−0.059	0.479
C6	37	0.508	<0.001	−0.028	0.755	0.078	0.389	−0.112	0.179
	**Curious**	**Intensity**	***P***	**Extensity**	***P***	**Pause**	***P***
**(B) Objective video-viewing pattern**							
C1	31	0.060	0.468	−0.028	0.753	−0.166	0.061
C2	61	0.119	0.147	−0.005	0.952	0.111	0.215
C3	58	−0.001	0.990	0.177	0.047	−0.053	0.559
C4	52	0.019	0.813	0.038	0.673	0.162	0.072
C5	47	0.109	0.180	0.022	0.803	0.237	0.008
C6	37	−0.010	0.905	−0.091	0.310	0.066	0.466

### Factors Associated With Short-Term Learning Outcomes in Multivariable Analysis

Table 3 displays the adjusted factors associated with short-term learning outcomes determined through summative assessments. Male gender, concepts that agreed with previous understanding (C1), concepts that aroused curiosity (C1, C4, C6), concepts that “were learnt most” (C5), and concepts that agreed with video watching patterns of extensity (C2, C6) and pause frequency (C1, C6), and providing comments were significantly associated with essay assessment scores. Concepts that agreed with previous understanding (C3), aroused curiosity (C1, C3), were learnt most (C1), and were associated with a request for more teaching (C3, C4) significantly correlated with case analysis assessment scores (Table 3). Specific curiosities for concepts 1, 4, and 6 and concepts 1 and 3 were significantly associated with scores for the essay question and case-based analysis, respectively. The effect of specific curiosities on short-term learning outcomes, however, was not positively associated for all factors. Factors related to concept 6 (curiosity, extensity, and pause frequency) were positively associated with essay scores. Factors related to concept 3 (curiosity, agreement with previous understanding, most learnt, and request for more teaching) were associated with case analysis scores.

**Table 3:** Variables associated with summative assessment of learning outcomes in multivariable general linear regression using backward selection.

**Essay question scores**	**Coefficient**	**Standard error**	***P***	**Case-based analysis scores**	**Coefficient**	**Standard error**	***P***
Male gender	−0.438	0.174	0.013				
**Agree with previous understanding**				**Agree with previous understanding**			
C1	0.285	0.099	0.005	C3	−2.606	1.216	0.034
C5	−0.120	0.066	0.072				
Difficulty	0.315	0.184	0.091				
**Curiosity**				**Curiosity**			
C1	−0.534	0.199	0.009	C1	7.233	2.963	0.016
C4	−0.332	0.167	0.049	C3	−4.890	2.439	0.047
C6	0.437	0.200	0.031				
**Most learnt**				**Most learnt**			
C5	0.358	0.165	0.032	C1	−8.705	2.798	0.002
Provide comments	0.409	0.177	0.022	C3	4.738	2.629	0.074
Online video watching pattern				Teach more			
Extensity				C3	−4.808	2.309	0.040
C2	−0.486	0.208	0.021	C4	5.447	2.276	0.018
C6	0.390	0.161	0.017				
Pause frequency
C1	−0.380	0.166	0.024				
C6	0.591	0.292	0.045				

### Student Feedback

Table 4 presents students' feedback after online video learning. Most common comments were coded in the category of “appreciation of considerate lesson preparation or course framework” followed by “general gratitude” and “good learning efficiency and self-reported outcome.” In the dimension of curiosity, “novelty” and inducing “interest and/or curiosity” were cited frequently. Although participants sensed an information gap after online video learning and asked for specific content, they were satisfied that the online video learning process was concise and clear. Some participants strongly recommended applying this framework to other courses.

**Table 4:** Students' feedback.

**Coding category**	**The number of code**	**Typical remarks and codes**
**General gratitude**	26	Terrific and thank you. (general)
**Infrastructure-wise**		
Appreciate considerate lesson preparation or course framework	49	Perfect pre-class framework arrangement which prepares us for advanced learning in in-person class without overloading us. (framework, load)
Pre-class loading concern	9	Adequate amounts of content and sparkle learning interest. (load, interest)
**Curiosity-wise**		
Novel feeling and enjoyable reflection	7	The framework design is very novel and considerate for students. Topics are interesting! I enjoyed this style of learning key concepts and inductive learning which induce learning interests and enhance learning efficiency. (framework, interest, novel, efficiency)
Inducing interests and curiosity	8	Wonderful videos that remind us the important concepts and induce our curiosity! (curiosity)
Concise and clear	16	Concise content with depth; grab the key points in a short time without distraction; great innovation! (concise, novel)
Query for specific contents (information gap)	13	Understandable talking! I am interested in the pathophysiology part after the online video-learning but feel that the content can be involved more and want to know more about relevant clinical management guidelines. I expect clearer explanations in in-person class! (narration, query)
**Learning outcome**		
Learn a lot and good learning efficiency	19	Easily absorbable and effective learning! (efficiency)
**Miscellaneous**		
Suggesting modifications	7	Thanks for kind consideration in preparing the course. May add an overview introduction to integrate the video pieces. (framework, modification)
Suggest other courses imitation	2	I feel this teaching module is great! Apply this framework to other courses would benefit medical students a lot! (framework, imitation)
Good narration	3	Beautiful and clear narration! (narration)

## Discussion

This study revealed 4 major findings. First, students spent a median of 1.6 h engaged in online activities, and most were satisfied with online video learning. Second, self-reported curiosity was individually associated with the most learnt concept, and pause frequency correlated with curiosity in certain concepts. Third, curiosity about studied concepts following the online video learning suggested various effects of variable sizes and directions on short-term learning outcomes. Finally, interpretation of the students' feedback reflected that the online video learning process cultivated curiosity.

### Balance of Information Gap and Cultivating Curiosity in Medical Education

We observed aroused curiosity in the current study of online video learning, which could be explained by information gap theory. Besides, epistemic curiosity is useful to learn new ideas and solve intellectual problems, which were probed by quantitative questionnaires (Supplementary Table 1) and summative assessments, respectively. Loewenstein et al. (24) found that epistemic curiosity was greatest when participants had partial knowledge of a particular subject rather than no knowledge or full knowledge, a finding supported by Litman et al. (25) and Kang et al. (26). However, methods of managing the knowledge gap and potential consequences of varying degrees of gaps in knowledge (For example, a great knowledge gap may induce a loss of overall interest, and a gap that is too narrow may trigger satiety) require further investigation. Moreover, the instructional content designer must adjust the cognitive loads of online videos while simultaneously producing the most efficient learning environment.

### Curiosity, Learning Motivation, and Learning Outcomes

Our study demonstrated that curiosity impacted short-term learning outcomes, with various effect sizes and directions, and led to an increased understanding of the role that certain intellectual, emotional, behavioral, physical, and social factors have in the student learning process and social development. The underlying causes and explanations that aroused curiosity to C1 was associated with scores of both essay and case base analysis were not clear. It may be attributed to the question selection or something different (e.g., a new concept to the students) as compared to other topics. Further study is needed to figure out the root cause. Nonetheless, we showed that sparkle curiosity was associated with the short-term learning outcome. A wide variety of studies on learning have revealed connections between non-cognitive factors or skills (e.g., motivation, interest, curiosity, responsibility, determination, perseverance, attitude, work habits, self-regulation, and social skills) and cognitive learning results (e.g., improved academic performance, test scores, information recall, and skill acquisition) (27). The impacts of these non-cognitive factors may be modified and diluted in an adult learning setting, especially that of medical education. Four dimensions of measurement (fear, assumptions, technology, and environment) for curiosity inhibitors, proposed by Hamilton in the Curiosity Code Index (28), can likely be applied to assess people working in clinical medical education.

### Curiosity Online vs. Offline

We surveyed the extent of curiosity aroused in students just after they participated in the online video learning process. Sanjay analogized curiosity as the hunger of the brain, finding it, thus, to be a main element of a student's learning process (29). To spark curiosity through the online platform and develop expertise, Sanjay proposed helping students enter a state of mind conducive to questioning by assigning projects or asking questions online and then taking advantage of face-to-face class time by coaching and fine tuning. Our blended design is consistent with Sanjay's strategy and may also apply to other subjects, as one participant commented (Table 4).

### Curiosity in Blended Learning

Blended learning is an educational process of the thoughtful integration of classroom face-to-face learning experiences with online learning experiences (30). Blended learning appears to be more effective than or at least as effective as non-blended instruction for knowledge acquisition in health professions (31, 32). When designed well, blended learning courses in medicine can facilitate students to improve themselves in self-learning, understanding, and problem solving, ultimately enhancing their learning efficiency (33–35). A potential pitfall of blended learning is the isolated nature of e-learning if the student interacts on an individual basis with the computer and not with a peer group, which can be overcome in part by online engagement through webinars and discussion boards (36). Moreover, the amount of work for the developers of the courses was much more than expected in the beginning and quite a big difference may exist in applying the concepts as proposed by different persons teaching the same course (36). Students may not be used to the new educational concepts and the success of a blended learning heavily relies on self-study phase of the students (37). In our study, 55.6% students preferred the through class style and hypothetically more students would prefer the concept style rather than thorough which is more spoon-fed. This observation might probably be associated with an overloaded and exhausting curriculum and time table, and the norm of transmitting knowledge and skills by teachers in Asian education culture (38). However, the online component is constantly being enhanced as new media technologies become available (36). The spread of blended learning during the COVID-19 pandemic has forced its widespread adoption and demonstrated its benefits to a large constituency (36). It is likely that it will remain a mainstay of healthcare education in the future (36). Clinical educators and instructional designers are encouraged to creatively cultivate curiosity into the blended learning approach by applying multiple strategies (39, 40).

### Limitations

Applying no strict curiosity scales of measurement [e.g., epistemic curiosity vs. perceptual curiosity (3) or intellectual interest vs. information deprivation of Litman's Epistemic Curiosity Scale] (5) in the surveys limited this study. Whether these scales, along with a number of other non-cognitive factors, can be objectively assessed for medical students who are inundated with extensive medical knowledge is unclear. One participant's feedback stated that “The survey is too long and reductant. Please be as concise as possible.” We conducted educational studies to help medical students improve their learning efficiency but did not intend to add to the loading burden. The effect of teaching in class was not controlled during the analysis, which was a possible confounder. Although the survey response was anonymous to the educators to minimize vulnerability issue between the participants, the study results might be biased by induced positive response. Through this study, we determined the role of cultivating curiosity in short-term learning outcomes in medical education. Additional work is needed to repeat this survey to get more respondents from classes of other topics. Expansion on the difference in using this approach to teach medical vs. other health care students are worthwhile. Further studies are required to validate our results.

## Conclusions

Curiosity can be stimulated through pre-class online video learning in medical education. Such learning may induce medical students to further examine, either online or offline, other concepts about which information gaps were perceived through the learning process. As widespread applications of online technology are recruited for learning, the shifting role of in-person contacts would be toward coaching and mentoring in the future (41).

Aroused curiosity after online learning was associated with short-term learning outcomes in a blended framework of precision medical education. Curiosity also correlated with some features of the learning process, such as the most learnt concepts and watching pauses. Creating a “procuriosity” culture within the learning environment would foster learning efficiency and promote enjoyable learning experiences in medical education. Further investigation is required to determine the effect of this feature on long-term knowledge retention and to guide clinical career choices and development.

## Data Availability Statement

The raw data supporting the conclusions of this article will be made available by the authors, without undue reservation.

## Ethics Statement

The studies involving human participants were reviewed and approved by Institutional Review Board of National Taiwan University Hospital. Written informed consent for participation was not required for this study in accordance with the national legislation and the institutional requirements.

## Author Contributions

C-MH drafted the manuscript. C-CY, R-HH, and P-HL designed the study. C-MH, J-YW, and C-CY conducted data processing. C-MH and J-YW performed data analysis. J-YW and R-HH were the directors responsible for general organization and instruction. All authors contributed to the article and approved the submitted version.

## Conflict of Interest

The authors declare that the research was conducted in the absence of any commercial or financial relationships that could be construed as a potential conflict of interest.

## Publisher's Note

All claims expressed in this article are solely those of the authors and do not necessarily represent those of their affiliated organizations, or those of the publisher, the editors and the reviewers. Any product that may be evaluated in this article, or claim that may be made by its manufacturer, is not guaranteed or endorsed by the publisher.
