# Supplementary material for: Curiosity in Online Video Concept Learning and Short-Term Outcomes in Blended Medical Education
Source: Front Med (Lausanne). 2021 Nov 5;8:772956. doi: 10.3389/fmed.2021.772956 (PMC8602070; doi:10.3389/fmed.2021.772956)
Supplement: Supplementary file 1 [file Table_1.docx]

**Supplementary Table S1.** Online Questionnaire Form

**Part I**

**Q1:** Compared with my previous understanding, my knowledge of the core concept (C) after watching the preclass self-learning online video is

C1： □ Totally changed

- Largely changed
- Half and half
- Mostly unchanged
- Totally unchanged

C2： □ Totally changed

- Largely changed
- Half and half
- Mostly unchanged
- Totally unchanged

C3： □ Totally changed

- Largely changed
- Half and half
- Mostly unchanged
- Totally unchanged

C4： □ Totally changed

- Largely changed
- Half and half
- Mostly unchanged
- Totally unchanged

C5： □ Totally changed

- Largely changed
- Half and half
- Mostly unchanged
- Totally unchanged

C6： □ Totally changed

- Largely changed
- Half and half
- Mostly unchanged
- Totally unchanged

**Part II**

**Q2:** Which learning video(s) mostly induce(s) my curiosity?

□C1, □C2, □C3, □C4, □C5, □C6

**Q3:** I want to learn more about which concept(s)?

□C1, □C2, □C3, □C4, □C5, □C6

**Q4:** I hope the teacher can talk or hold more discussions on the topic(s) of

- C1, □C2, □C3, □C4, □C5, □C6

**Q5:** My preferred class style for the upcoming in-person class is

□ Complete and thorough introduction

□ Concept-oriented and raise study interests

□ Discussion in class and create a learning experience

□ Self-learning and class presentation

**Part III**

I feel _____ of the preclass video learning

**Q6:** Loading

□Very light, □Slight light, □Just fine, □Slight heavy, □Very heavy

**Q7:** Difficulty

□Very easy, □Slight easy, □Just fine, □Slight difficult, □Very difficult

**Q8:** Satisfaction

□Very unsatisfied, □Slight unsatisfied, □Just fine, □Slight satisfied, □Very satisfied

**Part IV**

My comment and/or questions____________________________________
