# Supplementary material for: Curiosity in Online Video Concept Learning and Short-Term Outcomes in Blended Medical Education
Source: Front Med (Lausanne). 2021 Nov 5;8:772956. doi: 10.3389/fmed.2021.772956 (PMC8602070; doi:10.3389/fmed.2021.772956)
Supplement: Supplementary file 2 [file Table_2.docx]

**Supplementary Table S2**. Core Concepts of the ‘Acute Liver Failure’ Course

| 1. Acute liver failure is a systemic syndrome. |
| --- |
| 1. The international normalized ratio of prothrombin time is both a diagnostic and prognostic factor for acute liver failure. |
| 1. Blood urea nitrogen and phosphate are liver-related biomarkers in acute liver failure. |
| 1. Renal dysfunction due to hepatorenal syndrome is much less frequently observed than that due to dehydration, infection, or drug toxicity. |
| 1. Deterioration of hepatic encephalopathy can be reversed by correcting trigger/aggravating factors—usually infection, bleeding, or dehydration. |
| 1. Macrophage plays an vital role in body fluid status dynamic fluctuation during the disease course. |
